# Clinical and virological features of first human monkeypox cases in Germany

**DOI:** 10.1007/s15010-022-01874-z

**Published:** 2022-07-11

**Authors:** Sebastian Noe, Sabine Zange, Michael Seilmaier, Markus H. Antwerpen, Thomas Fenzl, Jochen Schneider, Christoph D. Spinner, Joachim J. Bugert, Clemens-Martin Wendtner, Roman Wölfel

**Affiliations:** 1 MVZ München am Goetheplatz, Munich, Germany; 2 Bundeswehr Institute of Microbiology, Munich, Germany; 3 School of Medicine, University Hospital Rechts der Isar, Technical University of Munich , Munich, Germany; 4 Munich Clinic Schwabing, Academic Teaching Hospital, Ludwig-Maximilian University (LMU), Munich, Germany; 5 German Center for Infection Research (DZIF), Munich Partner Site, Munich, Germany

**Keywords:** Monkeypox, Skin lesion, Efflorescence, Semen, Blood, Germany, HIV

## Abstract

**Background:**

Monkeypox is a zoonotic orthopoxvirus infection endemic in central and western Africa. In May 2022, human monkeypox infections including human-to-human transmission were reported in a multi-country outbreak in Europe and North America.

**Case presentations:**

Here we present the first two cases of monkeypox infection in humans diagnosed in Germany. We present clinical and virological findings, including the detection of monkeypox virus DNA in blood and semen. The clinical presentation and medical history of our patients suggest close physical contact during sexual interactions as the route of infection.

**Conclusion:**

Monkeypox requires rapid diagnosis and prompt public health response. The disease should be considered in the current situation especially the differential diagnosis of vesicular or pustular rash, particularly in patients with frequent sexual contacts. Most importantly, it is essential to raise awareness among all health professionals for the rapid and correct recognition and diagnosis of this disease, which is probably still underreported in Europe (Adler et al. in Lancet Infect Dis https://doi.org/10.1016/s1473-3099(22)00228-6, 2022).

## Introduction

We describe the first two cases of monkeypox (MPX) infections in Germany to highlight the importance of recent developments for health professionals worldwide and to share further observations related to human-to-human transmission in these cases. MPX is an orthopoxvirus infection that, with the exception of a few imported cases in the past, was previously thought to be endemic only in West and Central African countries [2, 3]. Beginning in 2018, the United Kingdom (UK) Health Security Agency has reported several imported cases associated with travellers from these countries [4]. One case reported on May 7, 2022 had a travel history to Nigeria. However, the two other cases within a family reported on May 13, 2022; neither had a travel history nor an epidemiological link to the previous case. On May 17, 2022, four additional UK cases were reported involving individuals who described themselves as belonging to the group of men who have sex with men (MSM). Subsequent testing for monkeypox virus (MPXV) in symptomatic MSM patients reporting to sexual health and sexually transmitted disease (STD) clinics in the UK and elsewhere revealed an increasing number of confirmed MPX cases in Europe [2].

## Clinical description

Patient #1, a 26-year-old MSM sex worker, currently residing in Portugal, first presented to a previously received a note from a man with whom he had sexual intercourse on May 9. The latter had informed him of a skin rash and suspected syphilis infection. The patient reported living with human immunodeficiency virus (HIV) infection since 2017 and is currently immunologically well controlled and stable on combination treatment with bictegravir, emtricitabine, and tenofovir alafenamide.

**Fig. 1:**
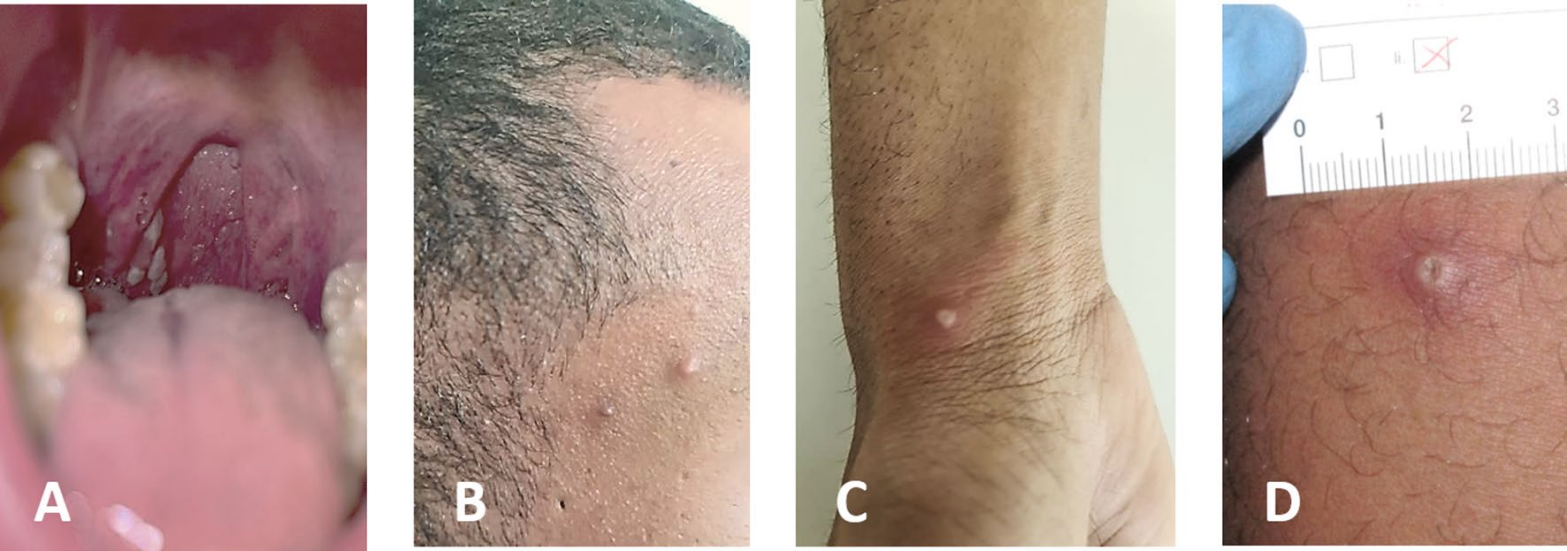
**A** Enoral lesions (right tonsil) visible already at first presentation of patient #1. **B**–**D** Both patients developed 10–12 initially vesicular, later pustular skin lesions distributed over the entire body. Many of these lesions were umbilicated, and all were at the same general stage of development. Upon opening of the lesions, the typical septate structure of pox lesions became apparent

On initial presentation, patient #1 complained of malaise since May 13, fever, mild joint, muscle, and back pain, and headache. He also reported dysphagia and the observation of white spots on his tonsils (Fig. 1A). Samples were collected for common STD screening. Two days later, patient #1 had developed several papular skin lesions on the trunk, extremities, and head (Fig. 1B). One lesion on the right wrist was particularly conspicuous (Fig. 1C). In light of recent cases in several other European countries, an MPXV infection was suspected. Swabs from pustules, as well as serum and plasma samples, were collected and sent to the Bundeswehr Institute of Microbiology (IMB) for MPXV-specific diagnostics. Local health authorities were notified about a suspected case of MPX.

Patient #2, a 32-year-old man, presented to his primary care physician on May 19, complaining of fever up to 39.0° Celsius, fatigue, and cough. Two days later, he developed inguinal lymphadenopathy, anal pain, and multiple vesicular skin lesions on the entire trunk (Fig. 1D). He reported having had repeated unprotected homosexual intercourse between May 1 and 17, 2022.

Both patients were transferred to the isolation ward of the Munich Clinic Schwabing, one out of seven Competence and Treatment Centres for highly contagious infectious diseases in Germany. The further course of disease to date was mild in both patients. No specific treatment was required other than topical zinc oxide suspension.

## Laboratory analysis

The virological screening at IMB for orthopoxvirus infections employs a molecular biological and serological algorithm using EN ISO 15189 accredited diagnostic methods. Patient samples were examined with a qPCR assay for simultaneous detection of all orthopoxvirus species (RealStar Orthopoxvirus Kit, Altona Diagnostics, Germany) and positive samples were further tested with an MPXV-specific qPCR [5].

**Fig. 2:**
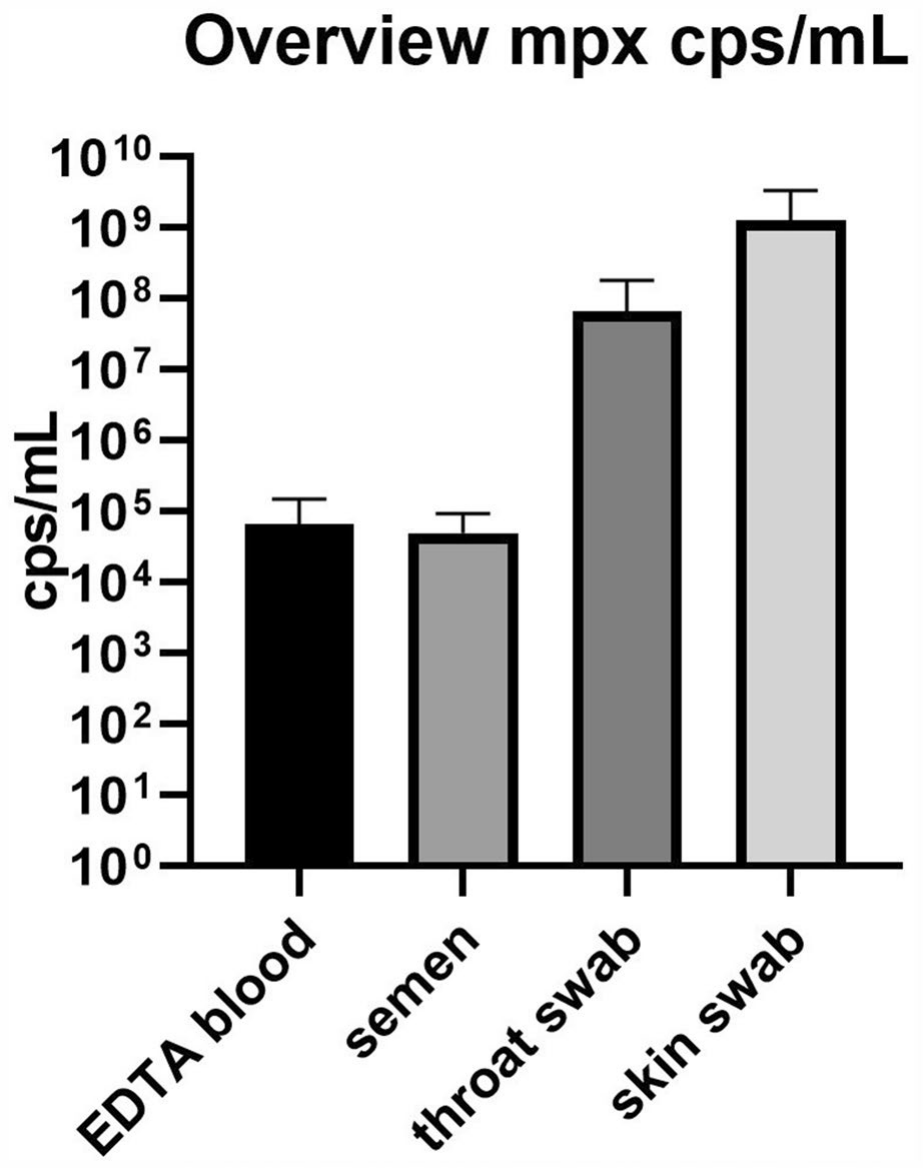
Histogram plot of MPXV genome copies per mL found in patient samples. Genome copies/mL of the two patients were averaged per sample type. Copy number per mL was determined using a linear dilution series of a quantified MPXV DNA standard

All swab samples tested positive in both qPCR assays (Fig. 2). Interestingly, blood samples also tested positive for MPXV DNA, indicating viremia in the respective stadium of infection as described before for MPX [6]. The known potential for MPX transmission through large oral droplets was confirmed by the detection of the virus in throat swabs of both patients. Notably, all semen samples showed positive MPXV qPCR results with viral DNA concentrations comparable to blood, while all urine samples tested negative. Highest viral concentrations were found in both patients in skin swabs from the pustules (Table 1 and Fig. 2).

**Table 1:** Overview of orthopoxvirus diagnostics from Patient #1 and Patient #2

Patient	Type of specimen	Date of sampling (2022)	qPCR results		Growth in cell culture (VeroE6)
			OPV cq values	MPXV cq values	
#1	Swab wrist pustule	19 May	20.15	27.2	Cpe after 2 dpi
#1	Swab head pustule	19 May	31.1	37.11	No growth
#1	EDTA blood	19 May	29.8	42.18	No growth
#1	EDTA blood	20 May	31.5	43.00	No growth
#1	Swab oral lesions	20 May	23.36	31.13	NA
#1	Skin swab	20 May	13.79	21.72	NA
#1	Skin swab	20 May	24.51	32.76	NA
#1	EDTA blood	22 May	Inhibition	44.15^a^	No growth
#1	Urine	22 May	Negative	Negative	No growth
#1	Swab oral lesions	22 May	17.39	25.58	NA
#1	Skin swab	22 May	12.96	20.65	NA
#1	Semen	22 May	28.24	37.09	No growth
#2	EDTA blood	23 May	27.26	36.17	No growth
#2	Skin swab	23 May	18.28	25.05	NA
#2	Semen	24 May	30.27	44.96	No growth
#2	Urine	24 May	Negative	Negative	No growth
#2	Swab oral lesions	24 May	29.11	38.52	NA

*NA* not applicable due to inactivating compounds in the original material (eNAT™ medium); *cpe* cytopathogenic effect, *dpi* days post-infection, no growth: cell cultures were incubated for 7 dpi and were monitored visually and by MPXV qPCR.

^a^Inhibition: cq values not reliable due to inhibitory compounds in the original material; therefore, this material was excluded for calculation of copies/mL

**Fig. 3:**
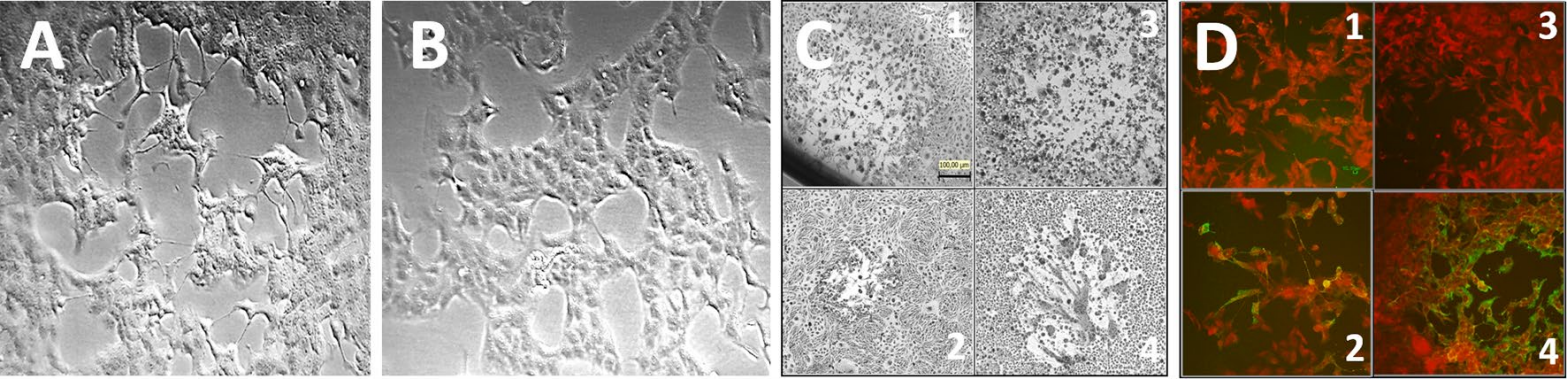
Isolation of MPXV-IMBmuc1 on Vero E6 cells and serology results. VeroE6 cells were inoculated with sample material from a lanced pustule of Patient #1 following standard procedure [17]. **A** Typical plaque formation of MPXV IMBmuc 1 on Vero E6 2 days post-infection of the sample material. **B** Vero E6 mock infected. **C** Plaque morphology of vaccinia virus (VACV) Elstree (1), VACV WR (2), MPXV IMBmuc 1 (3; most similar to VACV Elstree), MPXV IMBdrc 2510 (4), on MA104 cells 3 dpi—size bar refers to (1–4). **D** Reactivity of VACV Elstree in immunofluorescence assay with sera from patient #1 at hospital admission (1; non-reactive) and 11 days later (2; reactive, titre 80), sera of MVA unvaccinated (3; non-reactive), and MVA vaccinated (4; reactive) controls

Swabs from pustules, EDTA blood, and semen were used for simultaneous virus isolation in Vero E6 cell cultures. After 2 days, cell cultures from pustule material showed a cytopathogenic effect typical of orthopoxviruses (Fig. 3A), which was confirmed by MPXV qPCR. This indicates that a sufficient amount of infectious virus was present in the pustules to infect cells in culture and thus to be passed on by smear infection. All other cell cultures of patient #1 (semen, plasma, and urine) remained negative.

On the day of hospital admission, IgG antibodies against orthopoxviruses were not detectable in both patients by an immunofluorescence assay using vaccinia virus-infected MA104 cells, indicating a suboptimal or still developing antibody response. After 2 weeks, on May 30, Patient #1 showed seroconversion with an orthopoxvirus IgG antibody titre of 1:80 (Fig. 3D).

**Fig. 4:**
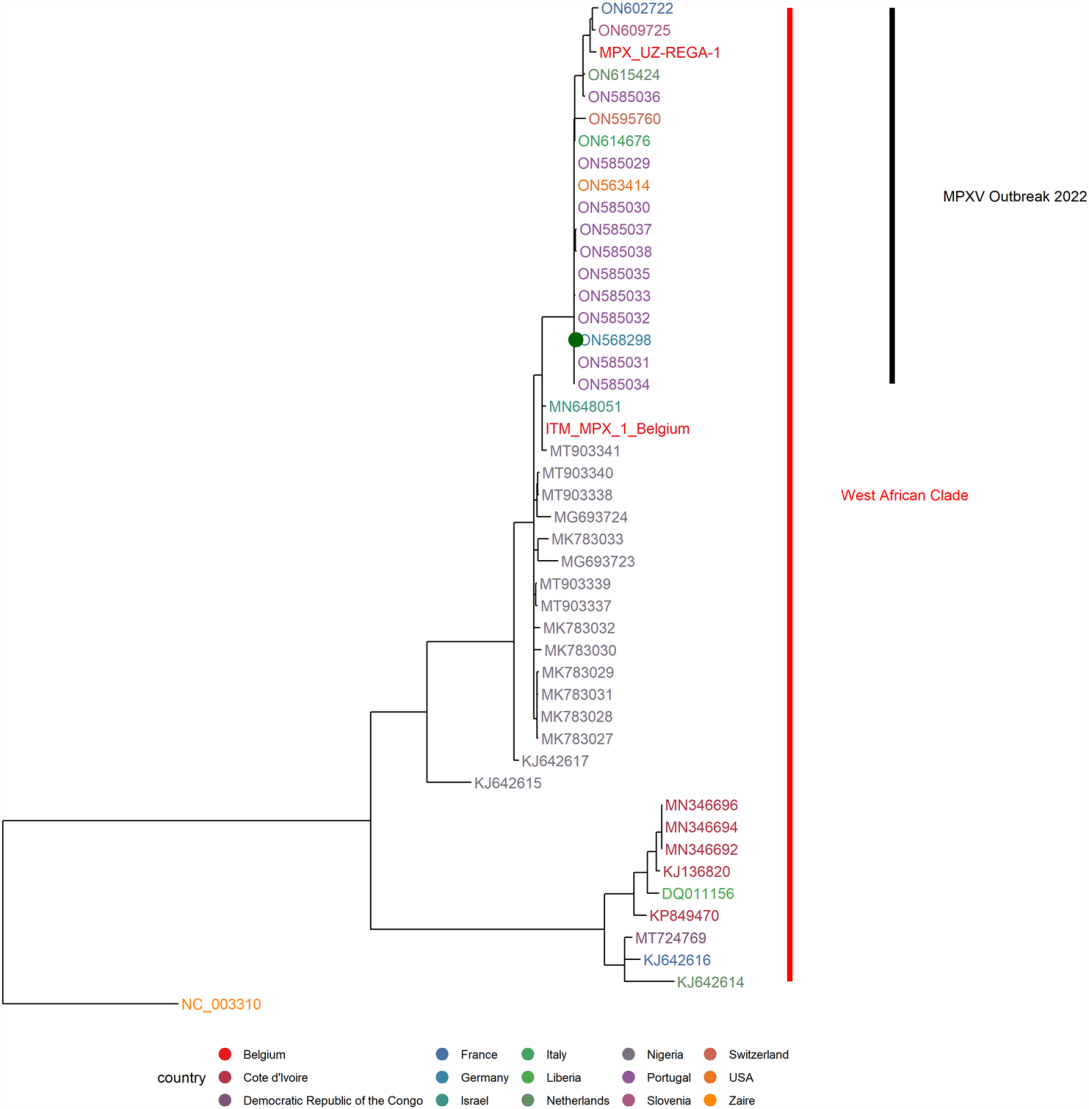
Maximum-likelihood tree based on selection of full genome sequences of MPXV. Genome sequence of MPXV-IMBmuc1 was obtained from direct sequencing of clinical material using Illumina-short read technology and subsequent de-novo assembly. A selection of full genomes of MPXV classified as West-African-clade as well as isolate Zaire-96-I-16 were used for alignment of this new sequence with phylogenetic grouping. The calculated tree was rooted at the separation between the Central and West African clades. German patient sequence marked with a green dot

A summary of the molecular and serological results obtained in both patients is shown in Table 1 and Figs. 3 and 4.

As with other previously reported cases, analysis of the Illumina sequenced viral genomes of patient #1 (AccNo. ON568298) resulted in an assignment to the West African MPXV clade. (Fig. 4). Both show genetic relationship to sequences from Portugal, Belgium, and the USA sampled in May 2022, but differ markedly from UK isolates of the same clade from the 2018 outbreak [7].

## Discussion

The described first two cases of MPX in Germany follow the pattern of recent MPX cases observed in North America and Europe. Most of them have not been associated with travel to countries where MPX is known to be an endemic zoonosis [8–10]. While in the past, transmission to humans was mainly through infected animals [11], current cases are predominantly, but not exclusively, transmitted between MSM.

As already known, close physical contact seems to be the main factor for human-to-human transmission. However, our clinical and virological investigations of the first two human MPX cases in Germany have revealed further findings that may be important for the monitoring and assessment of the ongoing MPX outbreak.

Interestingly, the oral lesions on the tonsils in patient #1 appeared before the development of other skin lesions. The site of manifestation could be related to oral intercourse reported by the patient in the sense of a primary affection by MPXV. On the other hand, involvement of lymphoid tissue in MPXV infections has been previously described in animal models [12]. Since enanthem-like oral mucosal lesions occur in many human viral infections, including SARS CoV-2 [13], it seems important to be generally alert for pharyngeal changes and to investigate them when typical skin lesions are not yet visible. In addition, the oral, genital, and anal lesions observed in the current multinational outbreak should be investigated more broadly in the light of possible sexual transmission.

The detection of MPXV DNA in semen, which we describe here for the first time, has never been reported for human MPX before. However, it is known from animal studies in mice that replicating vaccinia virus exhibits tropism for testicular and ovarian tissue [14, 15]. In our studies, we were able to isolate MPXV only from the contents of skin pustules. This confirms the importance of close (skin) contact as the main route of transmission. Other, as yet unknown, routes of transmission of MPXV to humans should nevertheless be further investigated.

Of note is also that the skin disease of the initial sexual partner of patient #1 was apparently not brought into connection with MPX, but with syphilis. Unfortunately, it remained unclear whether this individual was also tested for an MPXV infection later on. This observation underlines the importance of raising awareness of MPX as a new differential diagnosis of skin efflorescences in individuals who have not travelled to Africa. The emerging changes in the epidemiology of MPX [16] should be taken into account to properly identify individuals with mild and atypical clinical presentations of this human-to-human transmitted disease and to prevent its further spread.
